# Incidence of Neonatal Abstinence Syndrome (NAS) in Castilla y Leon (Spain)

**DOI:** 10.3390/children9010025

**Published:** 2021-12-30

**Authors:** Miriam Moreno-Ramos, Mercedes Sánchez-Barba, Rubén García Sánchez, José Antonio Mirón-Canelo, Veronica González-Nuñez

**Affiliations:** 1 Faculty of Medicine, University of Salamanca, 37008 Salamanca, Spain; mirianmoreno@usal.es; 2 Department of Statistics, Faculty of Medicine, University of Salamanca, 37008 Salamanca, Spain; mersanbar@usal.es; 3 Institute of Biomedical and Diagnostic Sciences (IBSAL), 37007 Salamanca, Spain; 4 Department of Pediatrics, University Hospital of Salamanca, 37008 Salamanca, Spain; Rubennigue@hotmail.com; 5 Department of Biomedical and Diagnostic Sciences, Faculty of Medicine, University of Salamanca, 37008 Salamanca, Spain; 6 Instituto de Neurociencias de Castilla y León (INCYL), University of Salamanca, 37008 Salamanca, Spain; 7 Department Biochemistry and Molecular Biology, Faculty of Medicine, University of Salamanca, 37008 Salamanca, Spain

**Keywords:** Neonatal Abstinence Syndrome (NAS), substance use disorder, opiates, Castilla y León, correlational research, biplot

## Abstract

Introduction: Neonatal Abstinence Syndrome (NAS) is considered a Public Health problem that is defined as a group of symptoms that appear in the newborn due to withdrawal from intrauterine drug exposure. Objective: The aim of this study was to analyze the incidence of NAS in Castilla y León from 2000 to 2019. Methodology: Data of NAS cases in Castilla y León from 2000 to 2019 were obtained. NAS incidence per 1000 births was calculated and the correlation among data from different provinces and years was analyzed. Results: The cumulative incidence of NAS in Castilla y León per 1000 births between 2000-2019 was 0.91‰, with great interprovincial variability. The provinces of Zamora and Palencia stand out, with high incidence rates of NAS despite their low birth rates. The temporal trend points towards a decrease in incidence from 2000 to 2019. Opioids such as methadone, cannabis, benzodiazepines and poly-drug use are the most prevalent drugs causing NAS, and it has also been observed that methadone is being replaced by cannabis as the major cause of NAS cases at the University Hospital in Salamanca in recent years. Conclusions: The incidence of NAS in Castilla y León decreased in 2004 and remained constant until 2019, but it shows great interprovincial variability. It is necessary to implement a national NAS Registry to obtain comprehensive information regarding its incidence.

## 1. Introduction

Substance use disorders occur when drug use causes a dysfunction, including health issues, and physical, psychological and/or social incapacity or disability. As a consequence, the subject fails to assume responsibilities at work, school or at home [1]. According to the World Drug Report published by the UN, in 2018,269 million people in the world consumed drugs. In addition to this, 35 million people worldwide suffered from illicit or legal drug use disorders, resulting in a negative impact in individuals, families and communities [2].

One third of drugs users are women [2] and, as a consequence of a substance use disorder, they show increased vulnerability and risk of suffering from mental health, and sexual and reproductive health disorders, as well as domestic and social violence [2]. In fact, there is a complex relationship between substance use disorder onset, violence and mental health. These issues can also lead to substance use disorders. Females who use drugs tend to be of childbearing age, and some are pregnant women [3,4]. The use of toxic substances during pregnancy places both the pregnant woman and the foetus at risk [5]. Substances taken by the mother can cross the placental barrier and reach the foetus, which can become intoxicated and passively dependent to the substance. At birth, the drug supply to the foetus stops abruptly, so that the newborn can develop a withdrawal condition known as Neonatal Abstinence Syndrome (NAS) [4,5]. Prenatal drug exposure also causes short, medium and long-term consequences [6,7]. Opioid drug use during pregnancy is associated with the development of congenital anomalies, intrauterine growth restriction, an increased risk of preterm birth and neurodevelopmental abnormalities [6] that may cause long-term functional impairments [7,8,9]. Maternal behavioural risk factors and the potential economic, social, nutritional, physical and mental issues of women using drugs during pregnancy may worsen global child health [9]. The mother’s surrounding environment and gender discrimination also play important roles in determining the mother’s opportunities and vulnerabilities.

### 1.1. Neonatal Abstinence Syndrome (NAS)

NAS is a group of signs and symptoms that appear in the newborn between 0-21 days of age due to withdrawal from intrauterine drug exposure. The onset of heroin withdrawal usually starts within 24 h of birth, whereas in the case of methadone withdrawal symptoms appear between days 1 to 3. In the case of non-opioid drugs, the onset is highly variable, as caffeine withdrawal can start at birth and chlordiazepoxide may start at day 21 [6]. NAS is clinically diagnosed, and both the clinical picture and its severity are conditioned by the substance ingested by the mother. NAS is generally a multisystemic clinical outcome that mainly affects the central and autonomic nervous system, the musculoskeletal system and the gastrointestinal tract. Irritability is a key symptom, although tremors, excessive crying, diarrhoea or even convulsions can appear. Although NAS rarely results in neonatal death, it usually leads to prolonged hospital stays [6,10,11], although the latter are related to the hospital policy [12].

### 1.2. Aetiology of NAS

A clear distinction should be made between NAS produced as a result of withdrawal from intrauterine drug exposure after birth and iatrogenic NAS that occurs after the medical use of certain substances, such as sedatives or anxiolytics, in the therapeutic management of newborns with other pathologies [6]. This work is focused on the first category, a syndrome incidence of which is clearly linked to the risk of substance use disorder in certain areas and societies.

NAS was firstly studied in newborns of pregnant mothers with opioid use disorders, so that the clinical picture related to opioids was closely linked to NAS [10,13]. Initially, NAS assessment scales were developed for the assessment of opioid use, and not for the use of other drugs, such as stimulants [14]. At present, NAS is a wider concept which comprises withdrawal signs and symptoms from both opioid and non-opioid drugs, whether legal or illegal. Thus, opioid withdrawal syndrome, which is specific for opioids and clinically more relevant, is separated from NAS produced by a wide diversity of drugs [15]. In this regard, there is controversy whether the neurobehavioural disorders observed in newborns exposed to non-opioid drugs, and which include signs such as irritability, tremors, fever or feeding disorders, may be due to a true withdrawal syndrome as a consequence of substance deprivation, or to substance intoxication of the newborn [6]. Another important consideration is maternal polydrug use, which modifies the clinical expression of NAS and is associated with greater severity [10,13]. Although NAS manifestations are similar for all drugs, each drug may have its special features in relation to the incidence of signs in the foetus, the time it takes for symptoms of appear (varying from hours to weeks, and even varying within drugs of the same pharmacological class), the duration of the clinical manifestations [6,10,13] and the long-term effects on the child [7,16,17]. Besides, there are several factors that may increase the severity and/or intensity of NAS [10,15]. As stated before, opioid NAS is the most prevalent syndrome, and 55–94% of newborns exposed to opioids in utero will develop withdrawal symptoms, although these percentages depend on the type of opioid [6,17,18]. In the case of non-opioid drugs, these percentages may be more variable [6].

### 1.3. Diagnostic Approach

Two methods have been used to identify prenatal exposure to drugs: the acknowledgement of drug use by the pregnant woman and the detection of substances in biological samples such as meconium, urine and hair; however, neither of these is the gold standard. The use of umbilical cord tissue for the analysis of drugs can also be a suitable detection method [6].

To standardise medical assessment of the newborn, and to determine and evaluate therapeutic management, different scales are used. According to a survey conducted in 2013, the most widely used scale is the Finnegan Neonatal Abstinence Scoring Tool (FNAST), which was developed by Dr. Loretta Finnegan in 1975 and is based on 21 clinical signs of withdrawal [19]. Therapeutic management is based on this nonvalidated scale, which was created to assess opioid NAS in full-term infants [12,20]. The original scale was modified to reduce redundancies, and the modified Finnegan Scale (M-FNAST) is the scale recommended by the American Academy of Paediatrics [21]. The M-FNAST classifies NAS according to a score (mild ≥8–11, moderate ≥12–15 and severe ≥16) [22], and also describes its therapeutic management. Other NAS scales have also been proposed: the Lipsitz Neonatal Drug-Withdrawal Scoring System (1975), the MOTHER NAS Scale (2010), the Finnegan Neonatal Abstinence Syndrome Tool Short Form (FNAST short form, 2013), the ESC model of care (Eat, Sleep, Console, 2018) which decreases pharmacologic interventions, and the NASCORE, a new NAS score (2019) [12,21,23,24,25]. However, none of these scales is universally adopted and all of them include some subjective items [23].

### 1.4. Therapeutic Management

Regarding the therapeutic management of NAS, up to now there has been no standardised clinical protocol in Spain and patient management is based on the published literature related to this pathology [26]. Although there is no standardized treatment for NAS, there are several therapeutic approaches, both pharmacological and nonpharmacological management [26]. Nonpharmacological interventions comprise the basic treatment of NAS [4,6,8]: monitoring the newborn, establishing the onset of symptoms, feeding on demand with hypercaloric formulas or breastfeeding (although the latter is a controversial issue), and rooming-in and placing the child in a quiet environment with few stimuli [10,27]. Pharmacological management depends on the score obtained in the assessment scales, which reflects the severity of NAS [4]. Opioid treatment, either morphine or methadone, is recommended by the WHO for opioid NAS [28]: some authors report that no significant differences have been obtained between these two drugs [29], whereas there are trials that have shown a difference in the two medications to treat NAS [30]. The WHO recommends the use of phenobarbital if NAS is caused by ethanol, sedatives or if the substance is not known, although the level of evidence is low [28].

The incidence of NAS in the US caused by fetal intrauterine drug exposure has increased considerably in recent years, from 1.2 per 1000 births (1.2‰) in 2000, to 7.3‰ in 2017 [31,32,33]. In 2014, one newborn was diagnosed with NAS every 15 min, which represents a total of 32,000 children [34].

According to data published in 2019 in the United States Annual Drug Use Surveillance Report, the highest rates of drug use are found among women of childbearing age, with the highest prevalence in age groups of 18 to 25, 26 to 34 and 35 to 39 years. NAS incidence rates are higher in infants born to mothers aged 18 to 25 [35,36], and 80% of the pregnancies in women who use drugs are unintended [36].The increased incidence of NAS is related to the increase in opioid use disorders among pregnant women, which quadrupled between 1999 and 2014, and also to the increase in opioid prescriptions in the US, which increased fivefold between 2000 and 2009 among the general population, including pregnant women [35,36].

Due to the high incidence rates of NAS in the US, it is considered a serious Public Health issue, and much research has been done on this pathology. There is deep knowledge on NAS aetiology, epidemiology and management, with established clinical protocols in the different states and in public and private health services [37,38]. The increased incidence and prolonged hospital stays (with an average length of 23 days) represent an annual cost of approximately 1.5 billion dollars for the US healthcare system [31]. On the other hand, in Spain there are very few public data regarding NAS incidence and aetiology, so that this problem remains invisible. The main aim of this work was to analyze the incidence of NAS in the Spanish region of Castilla y León, both global rates and per each of its nine provinces, in order to contribute to broaden the knowledge of NAS in Spain.

## 2. Materials and Methods

### 2.1. Geographic Scope and Objectives

Castilla y León is a Spanish Autonomous region that is administratively divided into nine provinces (Avila, Burgos, León, Palencia, Salamanca, Segovia, Soria, Valladolid and Zamora) with a total population of 2,401,307 inhabitants. In this study the null hypothesis is that NAS incidence in Castilla y León was similar with respect to NAS in all provinces and years between 2000 and 2019. We aimed to analyze the incidence of NAS in Castilla y León, to assess differences in the incidence of NAS among provinces and years.

### 2.2. Bibliographic Search

**Figure 1:**
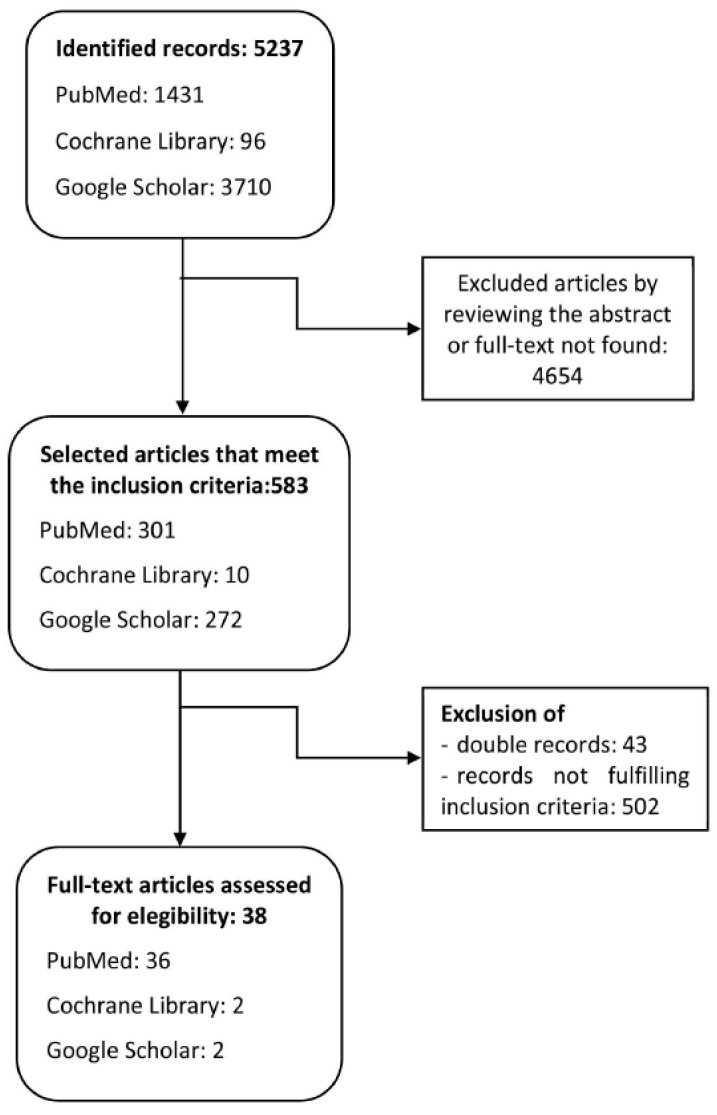
Flowchart of bibliographic research.

Although it was not our intention to conduct a systematic review, we performed a comprehensive bibliographic search on the incidence of NAS. A primary, secondary and tertiary literature review was conducted in different scientific databases and repositories between 20 October 2020 and 11 February 2021. Initially, PubMed (https://pubmed.ncbi.nlm.nih.gov/, accessed on 20 October 2020) and Cochrane Library (https://www.cochranelibrary.com/, accessed on 20 October 2020) and Google Scholar (https://scholar.google.es/schhp?hl=es, accessed on 20 October 2020) were searched with the inclusion criteria of full-text availability and adjustment to the proposed objectives. Systematic reviews and meta-analyses were prioritised, and repeated articles were excluded. The flowchart for the bibliographic search is depicted in Figure 1. Other international and national public databases were also searched (Table 1). Once the review was completed, we requested the data on the number of NAS cases in Castilla y León through the Transparency Website of Junta de Castilla y Leon (https://www.saludcastillayleon.es/transparencia/es; accessed date: 23 October 2020).

### 2.3. Data Analysis

Incidence of NAS in the nine provinces of Castilla y León was calculated for each year from 2000 to 2019, as well as the cumulated incidence and the total incidence in the entire region. Data were calculated with Microsoft Excel. Data normalization was achieved by dividing each of the calculated incidences by the cumulative incidence for the entire region (0.9123‰), so that incidence of NAS was transformed into an index (fraction of control). To quantify the contribution of each data to the total variability, data were treated as compositional data, which are vectors whose positive components represent proportions of a total, and consequently the sum of its components equals 1 [39]. Therefore, the number of NAS cases for each of the nine provinces in each of the studied years was divided by the total number of NAS cases in the entire region between 2000 and 2019. The same procedure was performed with the number of registered births, and the percentage of NAS cases for each province and year was divided by its corresponding percentage of births. This division allowed us to determine the contribution of each NAS case to the total number of cases, taking into consideration the variable “birth rate”. These data were used to build a Biplot [40,41] using R software.

## 3. Results

### Incidence of NAS in Castilla y León

**Figure 2:**
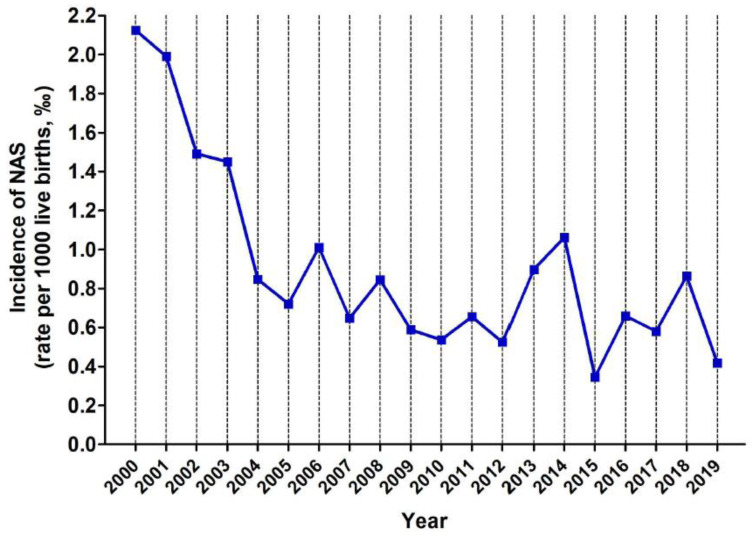
Incidence of NAS (expressed as rate per 1000 live births, ‰) in Castilla y Leon between 2000 and 2019.

**Figure 3:**
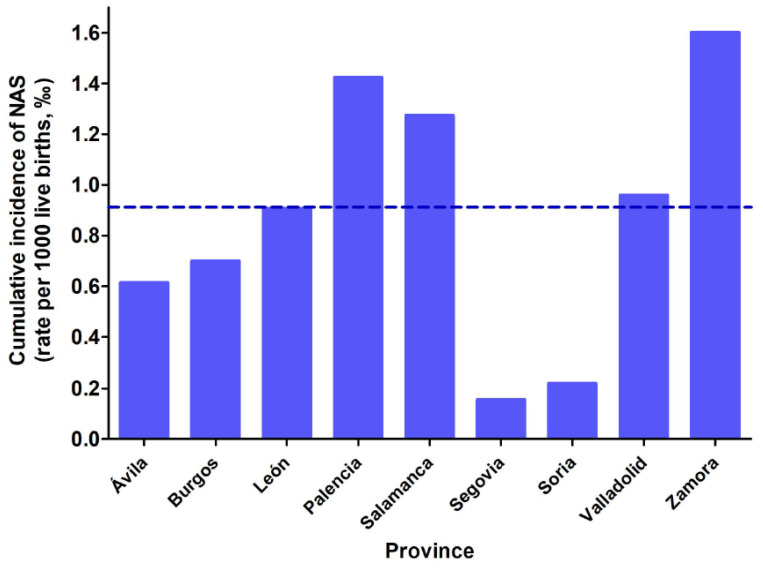
Cumulative incidence of NAS (expressed as rate per 1000 live births, ‰) in the nine provinces of Castilla y Leon between 2000 and 2019. The dashed horizontal line represents the cumulative incidence for the entire region between 2000 and 2019 (0.912‰).

Initially, the Transparency Website of Junta de Castilla y León was contacted to obtain information about incidence of NAS in our autonomous region, and we received an email with the total number of NAS cases between 2000 and 2019. We requested a breakdown of these data by province, which was sent in a subsequent email. The birth rate for each of the provinces, and for each of the studied years, were retrieved from the statistics web page at Junta de Castilla y León (https://estadistica.jcyl.es/web/es/estadisticas-temas/movimiento-natural-poblacion.html, accessed on 25 October 2021), the standardised incidence of NAS was calculated as the number of NAS cases per 1000 births per year for each of the nine provinces (Supplementary Table S1), the incidence of NAS for the entire autonomous region (0.912‰), the cumulative incidence for each of the studied years (Figure 2) and the cumulative incidence by province (Figure 3).

**Figure 4:**
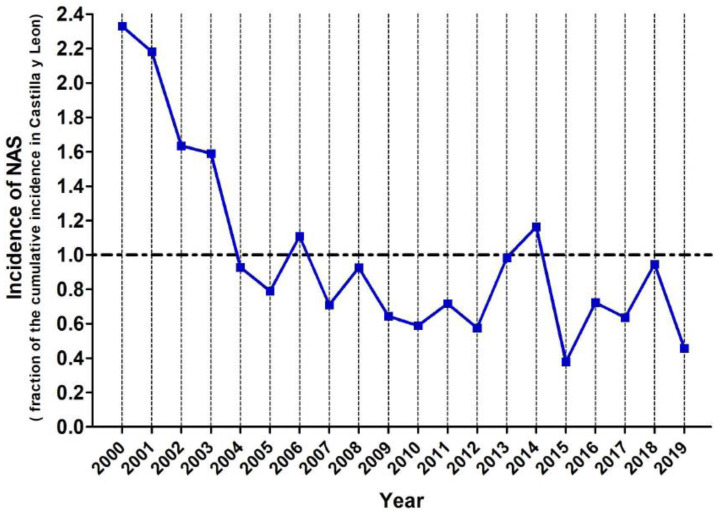
Normalized incidence of NAS (expressed as fraction of control ≡ cumulative incidence of NAS for the entire region) in Castilla y Leon between 2000 and 2019. The horizontal dashed line y = 1 corresponds to the cumulative incidence in the entire region, so that years with an index > 1 have higher incidence rates of NAS.

**Figure 5:**
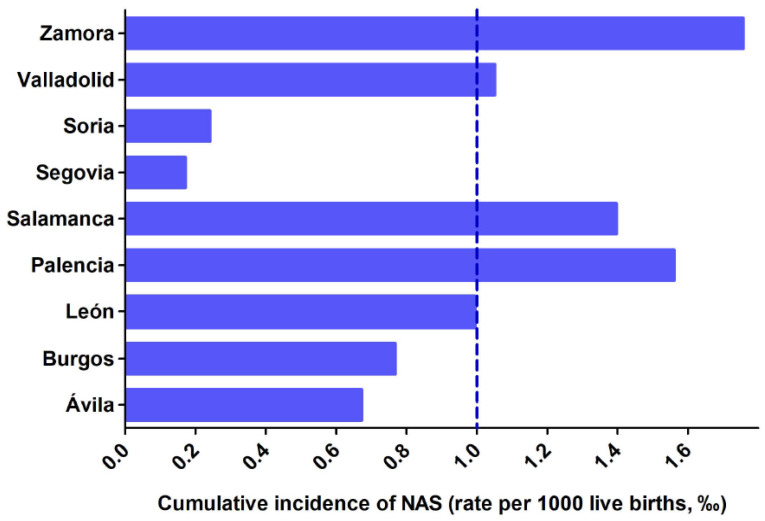
Normalized cumulative incidence of NAS (expressed as fraction of control ≡ cumulative incidence of NAS for the entire region) for each of the nine provinces of Castilla y Leon between 2000 and 2019. The vertical dashed line y = 1 corresponds to the cumulative incidence in the entire region, so that provinces with an index > 1 have higher incidence rates of NAS.

The mean incidence over the 19 years of study was 0.867 ± 0.961 new cases per year per 1000 live births (‰) and the cumulative incidence over the entire period was 0.912‰. Avila, Segovia and Soria, which have low birth rates and aged populations, had very few NAS cases, and the cumulative incidence for these provinces were, respectively, 0.614‰, 0.156‰ and 0.219‰. The more populated provinces had higher incidence rates of NAS: 0.700‰ in Burgos, 0.907‰ in León, 1.275‰ in Salamanca and 0.959‰ in Valladolid. Finally, Zamora and Palencia showed high incidence of NAS despite their low birth rates: 1.603‰ and 1.424‰ respectively. The analysis of the temporal evolution for the incidence of NAS showed a declining trend in the reported NAS cases from 2000 to 2004 and a stabilizing trend from 2004 to 2019 (Figure 2). To determine the provinces and the years with the highest incidence rates of NAS, data were normalised: the incidence of NAS for each province and for each of the studied years was divided by the cumulative incidence for the entire region. Thus, those years and provinces with an index > 1 had higher incidence rates of NAS than the cumulative incidence for the entire community (see Figure 4 and Figure 5 and Supplementary Table S2). As it can be seen, the first four years of this study (from 2000 to 2003) show high incidence indexes, albeit with a declining trend, and a stabilization is observed for the subsequent years. When data of the different provinces was analyzed, it was observed that Salamanca, Palencia and Zamora showed higher indexes, while Soria and Segovia were well below the average of the entire region.

**Figure 6:**
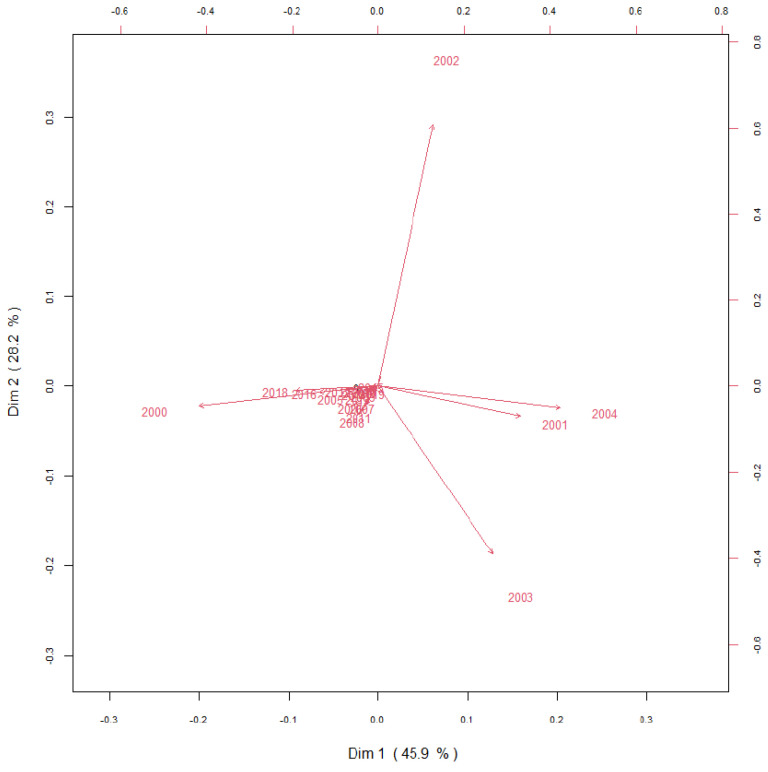
Covariance biplot of the compositional data of the incidence of NAS in Castilla y Leon for each year between 2000 and 2019.

**Figure 7:**
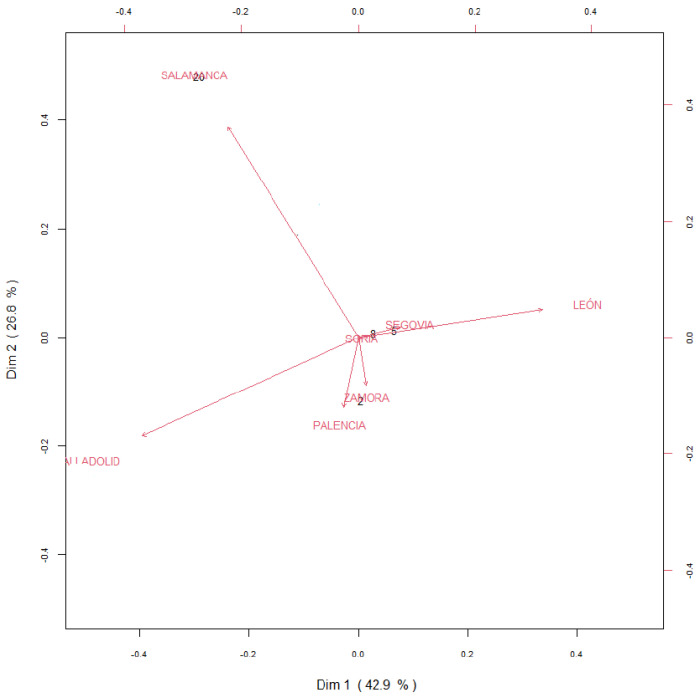
Covariance biplot of the compositional data of the cumulative incidence of NAS in the nine provinces of Castilla y Leon between 2000 and 2019.

To determine the contribution of each NAS case to the total incidence of NAS in Castilla y Leon, results were transformed into compositional data to perform multivariate analysis and Biplots were constructed for the variable “year” (Figure 6) and “province” (Figure 7). Biplots are frequently used to visualize the results of multivariate analysis. The observations are displayed as points in one plane, which is formed by two variables, one variable per axis. The origin is symbolized by O and the points are characterized by vectors, which are called arrows. The length of the vector represents the standard deviation of each variable, so that the arrow is longer when the variable displays high variability. The distance between two points is proportional to the standard deviation between variables. The cosine of the angle between two arrows approaches the correlation between a pair of variables: a sharp angle indicates positive correlation, a right angle indicates independent variables (no correlation) and an straight angle indicates a negative correlation. The variable “year”, data are homogeneous as they do not show high degree of variability (arrows are short and located at the origin of the biplot), except for the time period 2000 to 2004. The year 2001 strongly correlates with 2004, and these two years show a certain degree of positive correlation with 2003; however, 2001 and 2004 inversely correlate with 2000 and show independent trends when compared to 2002. The years 2002 and 2003 are inversely correlated. The analysis of the variable “province” indicates that León, Salamanca and Valladolid show the highest degrees of variability. Salamanca shows an independent trend when compared to Valladolid or to Leon. Valladolid and Leon are inversely correlated, and Palencia and Zamora show positive correlation.

## 4. Discussion

### Current Status of NAS in Spain

According to the National Survey on Alcohol and Other Drugs in Spain (Informe EDADES, the National Drug Report), the most commonly abused drugs among women were alcohol (with a prevalence rate of 54%), tobacco (32%), hypnosedatives with or without prescription (11%), cannabis (5%) and cocaine (0.3%) [42]. Prevalence rates of hypnosedatives and opioid analgesics were higher in women than in men, and reports of drug use in women of childbearing age (15–44 years) revealed that only 19.46% of these women did not used any legal or illegal drug in the last year, whereas 44% used one drug and 36.46% were polydrug users (25.6% used two drugs, 8.4% used three drugs and 2.47% used four or more drugs) [42].

After reviewing the existing literature and databases, no institutional reports on the incidence of NAS in Spain were found, and only a few reported the incidence rate of NAS in hospital such as 2.65‰ in Hospital Materno-infantil in Badajoz in 2014 [43] and 7.5‰ in Cabueñes (Asturias) in 2011 [44]. The prevalence rate of substance use disorder during pregnancy in Spain is not institutionally recorded, and only are very few studies have been carried out in different Spanish hospitals. In 2009, a study conducted at the Hospital del Mar (Barcelona) revealed that maternal interviews underestimated substance use disorder in pregnant women [45]. The self-reported prevalence (as stated in interview sessions) was 1.5% for cannabis, 1.2% for cocaine and 0.3% for heroin, whereas the calculated prevalence rates by meconium analysis was 5.3% for cannabis, 2.6% for cocaine and 4.7% for heroin. Another study conducted at the Hospital Regional Universitario in Málaga in 2014 showed that the self-reported prevalence rates were 27.2% for alcohol, 15% for tobacco and 2.43% for cannabis [46]. Concerning the incidence of NAS in Castilla y León, the deposited information in public databases is relatively scarce and no data were found on the incidence of NAS, nor on the prevalence rate of drug use among pregnant women. According to Informe EDADES, the prevalence of drug use in Castilla y Leon is similar to the national trend [42]. The most commonly abused drugs among women in Castilla y Leon are alcohol (37.7%), tobacco (9%) and cannabis (0.3%), while heroin use is less than 1% [47]. In 2019 in Castilla y Leon, only 18.5% of the patients admitted to outpatient care for consumption of psychoactive substances were women [48], and 204 were admitted for illicit-substance use disorder [49]. A total of 335 women were treated with medication for opioid use disorder, 320 related to methadone (with an average age of first drug use of 23.6) and 15 related to buprenorphine treatment [48,49]. These results indicate that women of childbearing age are the most prevalent group with substance dependence and, due to the high addictive potential of the most prevalent drugs, it is very likely that these women continue consuming drugs during pregnancy, and hence their newborns will very likely develop NAS symptoms.

Our results indicate that the incidence of NAS varies among the nine provinces of Castilla y Leon, and that the provinces of Palencia and Zamora display high prevalence rates, despite their aged populations (approximately half of their population is over 50 years old [50]. It would be interesting to analyze the collected data on substance use disorder for each province to better interpret these results. Some of the possible explanations may be related to a larger proportion of pregnancies among young women (aged under 30) or the migration of qualified workers from rural to more urbanized areas in the search for better job opportunities. In both provinces there are centres for the treatment and prevention of drug dependencies among young people who use drugs (Proyecto Hombre Association). The opposite trend is observed in Segovia, Soria and Ávila, which have low incidence rates of NAS. Of note is that the incidence is 10 times lower in Segovia than in Zamora, two provinces with similar population size. This may be due to less illicit drug use in Segovia, or to under-diagnosis of NAS cases. In Soria, the birth rate is significantly lower than in the rest of the provinces, so that the incidence of NAS is also affected. In Avila the incidence of NAS was high in 2000, but as can be seen in this study, it has decreased and the cumulative incidence rate is 40% lower than the mean of the entire region. Valladolid and León show similar incidence rates of NAS; however, biplot analysis indicates that their data are inversely correlated.

The incidence of NAS in Salamanca is higher than the average incidence calculated for the entire region, although the percentage of aged population in Salamanca is higher than in Valladolid. It should be noted that Salamanca is a university city with more than 45,000 students from all over the country, as well as many international exchange students, which account for one third of its total inhabitants. Therefore, this young floating population influences its demography and socioeconomic activities, and may explain the high incidence rate of NAS. The Department of Pediatrics at the University Hospital of Salamanca (HUSA) has recorded cases of newborns diagnosed with NAS and their aetiology since 2008, and most of the cases are iatrogenic NAS caused by the use of fentanyl as sedative during surgery or in therapeutic hypothermia. Maternal methadone use is the most prevalent drug causing NAS in newborns, although there are a few cases of benzodiazepines, polydrug use and cannabis. Interestingly, the latter emerged in 2018 and 2019.

Regarding temporal trend, there was little variability among the studied years, except for the time period 2000 to 2004. The years 2000–2003 are those with the highest incidence rates (in the same order 2126‰, 1991‰, 1492‰ and 1451‰ respectively), but not with the highest interprovincial variability (Supplementary Table S3). This could be due to the lack of standardized clinical protocols, although consecutive years inversely correlate in the biplot. Among the potential explanations, we speculate that a similar trend in drug use occurred in 2001 and in 2004 as a result of stressful events such as the terrorist attacks to the Twin Towers of New York City’s World Trade Center on 11 September 2001, and on 14-M in Madrid in 2004.

In the USA, the Agency for Healthcare Research and Quality (HCUP from the AHRQ) keep a record of all cases of NAS, and the National Survey on Drug Use and Health (NSDUH) monitors the prevalence of drug use among pregnant women [1,3,33]. Thus, we can compare our data of the incidence of NAS in Castilla y León, which might reflect the Spanish National trend, with those data from USA (Table 2 and Table 3). Large differences are observed between the incidence of NAS in the USA and in Castilla y León, which may be related to high consumption of opioid analgesics in the USA (Table 4) and differences between the American and Spanish Health Care System. The Spanish National Health Care System is characterised by universal accessibility and a free-of-charge health coverage scheme that allows for prevention and health promotion among vulnerable populations, as is the case with people who use drugs. Besides, epidemiological differences in drug use between Spain and the USA may condition the incidence of NAS, especially the use of opioid analgesics. The so-called “opioid crisis” in the US since the late 1990s [51]) is of note as well as differences in the most commonly used opioids, i.e., combinations of NSAIDs and opioids in Spain (such as Tramadol/Paracetamol or acetylsalicylic acid and codeine [52], while hydrocodone and oxycodone are the most frequently prescribed opioids in the US [53].

**Figure 8:**
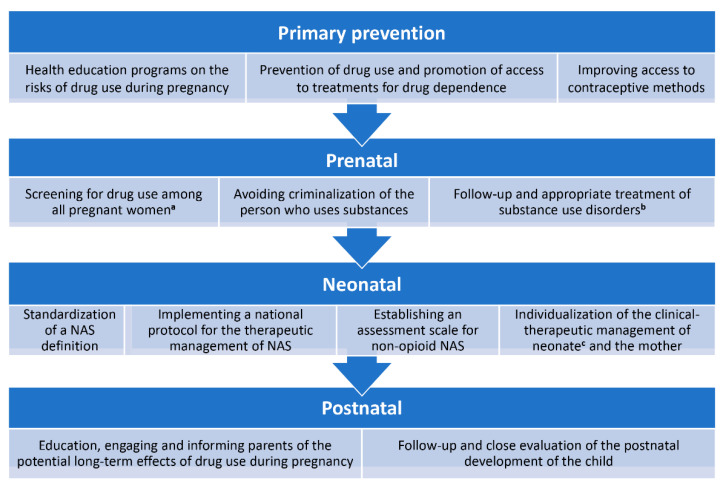
Clinical flowchart for a comprehensive management of NAS. The WHO strongly recommends healthcare professionals to ask pregnant women about past and present use of alcohol and other substances in the first and in all consecutive prenatal appointments [28]. The American College of Obstetricians and Gynecologists recommends conducting drug screening tests with validated questionnaires, such as the 4Ps questionnaire, NIDA Quick Screen and CRAFT [54], although they usually underestimate substance use and the real incidence of substance use disorder tends to be higher than in self-reported data [55,56]. Legend: ^a^: data taken from [4,10,12,29]; ^b^: accepted medical treatment during pregnancy includes methadone or buprenorphine, in addition to proper obstetric care and behavioral therapy [28,54,56]; ^c^: novel initiatives for the clinical management of NAS have been recently proposed that significantly reduce hospital stays and medication use [12].

This work aims to broaden the perspectives on both prenatal and postnatal clinical management of newborns with NAS and their mothers, with special emphasis on the follow-up of affected infants with NAS. Studies on the prevalence of drug use in pregnant women are of great interest, as they enable preventive measures. These studies could be carried out by introducing a specific section for women of childbearing age and pregnant women in the National Survey on Drug Use and Health in Spain, as well as by monitoring drug use in routine examinations during pregnancy, always under strict confidentiality. A study carried out at the University of Malaga proposed a questionnaire on drug use with individually validated questions [46]). Thus, we propose a comprehensive flowchart for the clinical management of NAS, from primary prevention to postnatal life of the affected child (Figure 8). In addition, social measurements should be taken into consideration to help the affected family.

The limitations of our work include the lack of data and lack of accessibility at the national level. Thus, our analysis is restricted to the Autonomous Community of Castilla y León, which is the largest but not the most populated region of Spain. Besides, Castilla y León has the most dispersed and aged population in Spain, mostly living in rural areas, which implies fewer NAS cases and, therefore, a reduction in its incidence. These considerations may condition and underestimate the national incidence of NAS, since higher incidence rates would be expected in other Autonomous Communities with younger populations. It seems that alcohol and tobacco are the most prevalent drugs used by people during pregnancy; given their potential to interfere with normal fetal development, it will be interesting to conduct an additional study about the incidence of negative health outcomes in newborns prenatally exposed to alcohol and tobacco. It would be advisable to generate a national database that includes the total number of NAS cases, their aetiology, risk factors, prevention and treatment. In Spain, registries are not generally available for the general public, as authorisation is needed to access this information. The data are blinded, do not include names or IDs, and it is not possible to identify the subjects. The personal data for each patient are only kept in the medical records, and it is not possible to access this information without informed consent, and specific permission of a Bioethics Committee is necessary to conduct a study involving personal data. Thus, the initiative of implementing a national registry of NAS cases will contribute to standardizing protocols for the clinical management of NAS.

## 5. Conclusions

The incidence of NAS in Castilla y León decreased from 2.126‰ in 2000 to 0.847‰ in 2004, and remained constant until 2019, although it showed great interprovincial variability. The currently available data on the incidence rate of NAS in Spain are incomplete and insufficient for a more comprehensive study to explain the differences observed between years and provinces. It would be necessary to establish a National Registry in order to make an in-depth national analysis of the incidence of NAS and the prevalence of drug use during pregnancy with the aim of taking preventive measures.

## Figures and Tables

**Table 1:** List of public databases consulted in this study.

Institution	URL
World Health Organization (WHO)	https://www.who.int/ (accessed date: 3 November 2020)
European Monitoring Centre for Drugs and Drug Addiction (EMCDDA)	https://www.emcdda.europa.eu/emcdda-home-page_en (accessed date: 3 October 2020)
National Institute on Drug Abuse (NIDA)	https://www.drugabuse.gov/es (accessed date: 8 October 2020)
Plan Nacional sobre Drogas (PND) (Spanish National Drug Plan)	https://pnsd.sanidad.gob.es/ (accessed date: 8 October 2020)
European Academy of Paediatrics (EAP)	https://www.eapaediatrics.eu/ (accessed date: 17 November 2020)
American Academy of Pediatrics (AAP)	https://www.aap.org/en-us/Pages/Default.aspx (accessed date: 23 November 2020)
Asociación Española de Pediatría (AEP) (Spanish Academy of Pediatrics)	https://www.aeped.es/ (accessed date: 23 November 2020)

**Table 2:** Incidence of NAS (expressed as diagnoses of NAS at newborns hospitalized per 1000 live births, ‰). *: Rate of NAS in 2015 is only based on the first three quarters of data, due to changes in ICD coding (ICD-9-CM, Code 779.5: Drug withdrawal syndrome in newborn; ICD-10-CM P96.1: Neonatal withdrawal symptoms from maternal use of drugs of addiction). ^#^—Data taken from [33].

Year	U.S. ^#^	Castilla y León
2009	2.9	0.6
2010	4.0	0.5
2011	4.1	0.6
2012	4.8	0.5
2013	5.8	0.9
2014	6.5	1.1
2015	6.6 *	0.3
2016	7.0	0.7
2017	7.3	0.6

**Table 3:** Drug use in the last 12 months in Castilla y León, Spain and in the US, expressed as percentage of the total population. Legend: ^a^—Data for population aged 15–64 years; ^b^—Data for population aged 12 years and over; ^c^—Data for benzodiazepines and barbiturates; *—No data available. Data taken [3,42,47].

Type of Drug	Castilla y León ^a^		Spain ^a^		US ^b^	
	General Population	Women	General Population	Women	General Population	Women
Cannabis	10.8	5.0	10.5	6.3	17.5	14.8
Powder cocaine	2.1	*	2.5	0,9	2	1.5
Amphetamines/Speed	0.8	*	0.7	0.4	*	*
Methamphetamines		*	0.3		0.7	0.5
Hallucinogens	1.1	*	0.6	0.2	2.2	1.5
MDMA (Ecstasy)	1.6	*	0.9	0.4	0.9	
Inhalants	0.3	*	0.1	*	0,8	0.6
Heroin	0.0	*	0.1	*	0,3	0.2
Hypnosedatives without prescription ^c^	0.6	*	1.3	1.2	2.1	2.1

**Table 4:** Opioid use in the last 12 months in Castilla y León, Spain and in the US, expressed as percentage of the total population. Legend: ^a^—Data for population aged 15–64 years; ^b^—Data for population aged 12 years and over; ^c^—Data referred to benzodiacepines and barbiturates. *—No data available. Data taken from [3,42,47].

Opioid Analgesics	Castilla y León ^a^		Spain ^a^		US ^b^	
	General Population	Women	General Population	Women	General Population	Women
With prescription	9	11.3	7.1	8.1	30.0 ^c^	31.8
Without prescription	*	*	0.6	0.7	3.7	3.3

## Supplementary Materials

The following are available online at https://www.mdpi.com/article/10.3390/children9010025/s1. Table S1: Incidence of NAS (expressed as rate per 1000 live births, ‰) in the nine provinces of Castilla y Leon and the total incidence in the entire region between 2000 and 2019. Table S2: Normalized incidence of NAS (expressed as fraction of control ≡ cumulative incidence of NAS for the entire region) in the nine provinces of Castilla y Leon between 2000 and 2019. Table S3: Average incidence of NAS in Castilla y León (average ± S.D. incidence of NAS in the nine provinces), expressed as rate per 1000 live births, ‰ between 2000 and 2019.
