# Supplementary material for: Incidence of Neonatal Abstinence Syndrome (NAS) in Castilla y Leon (Spain)
Source: Children (Basel). 2021 Dec 30;9(1):25. doi: 10.3390/children9010025 (PMC8774299; doi:10.3390/children9010025)
Supplement: Supplementary file 1 [file children-09-00025-s001.zip › children-1495250-supplementary.pdf]

Supplementary Table S1: Incidence of NAS (expressed as rate per 1000 live births, ‰) in the nine provinces of Castilla y Leon and the total incidence in the entire region between 2000 and 2019.

Year	Ávila	Burgos	León	Palencia	Salamanca	Segovia	Soria	Valladolid	Zamora	Castilla y león
**2000**	5,172	1,439	1,563	2,597	2,350	0,000	0,000	2,579	3,247	**2,126**
**2001**	0,887	1,123	2,509	1,717	2,023	0,806	1,488	2,803	2,681	**1,991**
**2002**	0,890	2,145	1,257	0,838	1,190	1,592	0,000	1,205	4,085	**1,492**
**2003**	0,000	1,065	1,236	3,322	2,303	0,000	1,355	0,926	4,122	**1,451**
**2004**	0,000	0,337	1,211	0,861	1,118	0,767	1,393	0,909	0,870	**0,847**
**2005**	0,756	0,654	0,906	1,633	0,760	0,000	0,000	0,437	1,724	**0,721**
**2006**	0,000	0,961	0,304	1,603	2,663	0,000	0,000	1,043	1,681	**1,011**
**2007**	0,000	0,000	0,853	1,656	1,083	0,000	0,000	0,633	1,748	**0,648**
**2008**	0,000	0,284	0,854	3,130	1,725	0,000	0,000	0,594	1,645	**0,845**
**2009**	0,000	0,604	0,861	0,812	1,503	0,000	0,000	0,203	0,869	**0,588**
**2010**	0,000	0,595	0,582	0,842	0,382	0,000	0,000	0,588	1,692	**0,537**
**2011**	0,000	0,000	0,000	2,445	1,132	0,000	0,000	1,472	0,000	**0,655**
**2012**	0,797	0,320	0,625	0,000	1,196	0,000	0,000	0,644	0,000	**0,525**
**2013**	0,000	1,000	0,986	1,742	0,442	0,000	0,000	1,622	0,000	**0,898**
**2014**	0,000	1,418	1,317	1,695	0,428	0,000	0,000	1,160	2,907	**1,062**
**2015**	0,000	0,362	0,330	0,000	0,427	0,000	0,000	0,477	1,032	**0,345**
**2016**	1,765	0,752	1,394	1,910	0,000	0,000	0,000	0,248	0,000	**0,660**
**2017**	0,000	0,815	0,000	0,929	0,986	0,000	0,000	0,544	2,212	**0,580**
**2018**	3,064	0,425	0,760	0,000	1,928	0,000	0,000	0,866	0,000	**0,864**
**2019**	0,000	0,000	0,422	0,000	1,552	0,000	0,000	0,608	0,000	**0,419**
**TOTAL**	**0,614**	**0,700**	**0,907**	**1,424**	**1,275**	**0,156**	**0,220**	**0,959**	**1,603**	**0,912**

Supplementary Table S2: Normalized incidence of NAS (expressed as fraction of control ≡ cumulative incidence of NAS for the entire region) in the nine provinces of Castilla y Leon between 2000 and 2019.

Year	Ávila	Burgos	León	Palencia	Salamanca	Segovia	Soria	Valladolid	Zamora	Castilla y león
**2000**	5,670	1,578	1,713	2,847	2,576	0	0	2,827	3,559	**2,330**
**2001**	0,972	1,231	2,751	1,882	2,217	0,884	1,631	3,073	2,939	**2,182**
**2002**	0,976	2,351	1,378	0,919	1,304	1,745	0	1,320	4,478	**1,635**
**2003**	0	1,167	1,355	3,642	2,525	0	1,485	1,015	4,518	**1,591**
**2004**	0	0,369	1,327	0,944	1,226	0,841	1,527	0,996	0,953	**0,928**
**2005**	0,829	0,717	0,993	1,790	0,833	0	0	0,479	1,890	**0,790**
**2006**	0	1,053	0,333	1,757	2,919	0	0	1,144	1,842	**1,109**
**2007**	0	0	0,936	1,815	1,187	0	0	0,694	1,916	**0,710**
**2008**	0	0,311	0,937	3,431	1,891	0	0	0,651	1,803	**0,926**
**2009**	0	0,662	0,944	0,890	1,648	0	0	0,223	0,952	**0,644**
**2010**	0	0,652	0,638	0,923	0,419	0	0	0,645	1,855	**0,589**
**2011**	0	0	0	2,680	1,240	0	0	1,613	0	**0,717**
**2012**	0,873	0,351	0,685	0	1,311	0	0	0,706	0	**0,576**
**2013**	0	1,097	1,080	1,910	0,484	0	0	1,778	0	**0,984**
**2014**	0	1,554	1,443	1,858	0,469	0	0	1,271	3,186	**1,164**
**2015**	0	0,397	0,362	0	0,468	0	0	0,523	1,131	**0,378**
**2016**	1,935	0,825	1,528	2,094	0	0	0	0,272	0	**0,723**
**2017**	0	0,894	0	1,019	1,081	0	0	0,597	2,425	**0,636**
**2018**	3,359	0,466	0,833	0	2,113	0	0	0,950	0	**0,947**
**2019**	0	0	0,463	0	1,701	0	0	0,667	0	**0,459**
**TOTAL**	**0,673**	**0,768**	**0,995**	**1,561**	**1,397**	**0,171**	**0,241**	**1,051**	**1,757**	**1,000**

Supplementary Table S3: Average incidence of NAS in Castilla y León (average ± S.D. incidence of NAS in the nine provinces), expressed as rate per 1000 live births, ‰ between 2000 and 2019.

Year	Incidence
**2000**	2,105 ± 1,614
**2001**	1,782 ± 0,768
**2002**	1,467 ± 1,140
**2003**	1,592 ± 1,409
**2004**	0,830 ± 0,432
**2005**	0,763 ± 0,611
**2006**	0,917 ± 0,937
**2007**	0,664 ± 0,719
**2008**	0,915 ± 1,067
**2009**	0,539 ± 0,525
**2010**	0,520 ± 0,539
**2011**	0,561 ± 0,908
**2012**	0,398 ± 0,440
**2013**	0,644 ± 0,716
**2014**	0,992 ± 0,984
**2015**	0,292 ± 0,344
**2016**	0,674 ± 0,809
**2017**	0,610 ± 0,737
**2018**	0,783 ± 1,067
**2019**	0,287 ± 0,526
